# P-1544. Evaluation of an FDA-Authorized AI Biomarker of Sepsis to Predict Mortality over Time in Patients Suspected of Infection

**DOI:** 10.1093/ofid/ofaf695.1724

**Published:** 2026-01-11

**Authors:** Lincoln C Updike, Carlos Lopez-Espina, Akhil Bhargava, Lee Schmalz, Shah Khan, Dennys Urdiales, Matthew Sims, Bobby Reddy, Nathan Shapiro, Gregory Watson

**Affiliations:** Prenosis, Chicago, IL; Prenosis, Chicago, IL; Prenosis, Chicago, IL; Prenosis, Chicago, IL; Prenosis, Chicago, IL; Prenosis, Chicago, IL; William Beaumont University Hospital, Corewell Health, Royal Oak, Michigan; Prenosis, Chicago, IL; Beth Israel Deaconess Medical Center, Boston, Massachusetts; Prenosis, Chicago, IL

## Abstract

**Background:**

After prompt antibiotics, fluid resuscitation, and source control, patient monitoring is essential to effective sepsis care. The Sepsis ImmunoScore is an AI biomarker authorized by the FDA for identifying patients at risk of sepsis. Previous studies have demonstrated its diagnostic and prognostic performance at baseline. In this study, we investigated the longitudinal prognostic value of the Sepsis ImmunoScore for predicting in-hospital mortality in patients at risk of infection across all hospital settings and compared it against available alternatives.

**Methods:**

In this prospective, multi-center study, we enrolled a subset of adult patients suspected of infection from 6 U.S. hospitals between Jan 2019 and Dec 2022. The primary endpoint was in-hospital mortality. To increase the event rate, we enriched for sicker patients. We assessed the relationship between patient mortality and daily Sepsis ImmunoScore measurements over the first 5 days following blood culture order using AUC, logistic regression, and Kaplan-Meier analyses. We also assessed the performance of the sequential organ failure assessment (SOFA), procalcitonin (PCT) and C-reactive protein (CRP) as comparators. All analyses accounted for the enriched survey design.

**Results:**

Among 600 enrolled patients, 49 (8.2%) died in the hospital and 551 (91.8%) survived to discharge. The Sepsis ImmunoScore was significantly higher in non-survivors at each timepoint. Logistic regression found statistically significant associations between the Sepsis ImmunoScore and mortality at each timepoint. The Sepsis ImmunoScore was also more predictive than comparators (e.g., AUROC: 0.88 on day 2 vs 0.81 (SOFA), 0.79 (PCT), 0.63 (CRP)).

**Conclusion:**

This prospective multicenter U.S. study found that the Sepsis ImmunoScore predicts patient survival over time and outperforms available alternatives. These results indicate a prognostic association that may be useful in monitoring patients for response to treatment, signs of deterioration or organ dysfunction, and hemodynamic instability.

Sepsis ImmunoScore has not been approved by the FDA for prognostic use over time, and the safety and effectiveness of Sepsis ImmunoScore for prognostic use over time has not been established.

**Disclosures:**

Lincoln C. Updike, BS, Prenosis: employee Carlos Lopez-Espina, MS, Prenosis: employee Akhil Bhargava, MS, Prenosis: employee Lee Schmalz, BS, Prenosis: employee Shah Khan, PhD, Prenosis: employee Dennys Urdiales, BS, Prenosis: employee Matthew Sims, MD, PhD, Affinity Biosensors: Grant/Research Support|Applied Biocode: Advisor/Consultant|Applied Biocode: Grant/Research Support|Astra Zeneca: Grant/Research Support|Biotest AG: Grant/Research Support|Inflarx: Advisor/Consultant|Janssen: Grant/Research Support|Leonard-Meron Biosciences: Grant/Research Support|Locus Biosciences: Grant/Research Support|Pfizer: Grant/Research Support|PhAST Corp: Grant/Research Support|Prenosis: Advisor/Consultant|Prenosis: Grant/Research Support|QIAGEN: Grant/Research Support|Seres: Advisor/Consultant|Seres: Grant/Research Support|Vedanta Biosciences: Grant/Research Support Bobby Reddy, Jr., PhD, Prenosis: Board Member|Prenosis: Ownership Interest Nathan Shapiro, MD, Prenosis: Advisor/Consultant Gregory Watson, PhD, Prenosis: employee

